# Morphology and genetics of *Lythrum salicaria* from latitudinal gradients of the Northern Hemisphere grown in cold and hot common gardens

**DOI:** 10.1371/journal.pone.0208300

**Published:** 2019-01-03

**Authors:** Beth A. Middleton, Steven E. Travis, Barbora Kubátová, Darren Johnson, Keith R. Edwards

**Affiliations:** 1 U.S. Geological Survey, Wetland and Aquatic Research Center, Lafayette, Louisiana, United States of America; 2 Department of Biology, University of New England, Biddeford, Maine, United States of America; 3 SoWa National Research Infrastructure, Biology Centre of the ASCR, České Budějovice, Czech Republic; 4 Cherokee Nations Technologies, Wetland and Aquatic Research Center, Lafayette, Los Angeles, United States of America; 5 Faculty of Science, University of South Bohemia, Ecosystem Biology, České Budějovice, Czech Republic; Washington University, UNITED STATES

## Abstract

The aim of this project was to compare the phenotypic responses of global populations of *Lythrum salicaria* in cold/dry and hot/humid environments to determine if phenotypic plasticity varied between the native and invasive ranges, and secondarily if this variation was linked to genetic diversity. Common garden studies were conducted in Třeboň, Czech Republic, and Lafayette, Louisiana, USA (cold/dry vs. hot/humid garden, respectively), using populations from latitudinal gradients in Eurasia and North America. *Lythrum salicaria* seeds collected from the same maternal plants across these latitudinal gradients were germinated and grown in Třeboň and Lafayette. Tissue masses (above-, below-ground, inflorescence and total) of these individuals were assessed at the end of each growing season (2006–2008). Worldwide field measurements of *L*. *salicaria* height were made by volunteers from 2004–2016. Biomass and height data were analyzed using the General Linear Model framework and multivariate techniques. Molecular markers (amplified fragment length polymorphisms) of individuals used in the common garden study were analyzed using traditional genetic diversity metrics and Bayesian clustering algorithms in STRUCTURE. Reaction norms were developed from differences in maternal plant responses in Třeboň versus Lafayette. In the common garden studies, stem/leaf, root and total biomass generally were highest for individuals grown from seeds collected in the southern part of the range in the cold garden, particularly by the third year of the study. In contrast, inflorescence biomass in the cold garden was higher by the third year in individuals from mid-latitude populations. As measured by volunteers, plants were taller in Eurasia than in North America moving from north to south with the pattern switching southward of 40°N latitude. Genetic diversity was similar between native and non-native invasive populations regardless of geographical origin of the seed and was not significantly different in the GLM Select model (p > 0.05). Reaction norm slopes showed that Eurasia had larger values than North America for reaction norms for above-ground and total biomass. Plants from the seeds of mother plants from Turkey had wide variation in total biomass when grown in Třeboň versus Lafayette; this variation in response within certain populations may have contributed to the lack of population-level differences in plasticity. These results indicate no loss of genetic diversity for *L*. *salicaria* during its North American invasion, nor reduction in plastic tissue allocation responses to a varying environment, which may help explain some of its invasive qualities and which could be of adaptive value under changing future environments.

## Introduction

Many studies have been conducted to explore the phenotypic expression of genetic traits of populations of species from various latitudes and/or continents using common garden studies [1], but few studies combine these outcomes with field collected data to gain a deeper understanding of the ecology and evolution of a species. A species with wide-ranging natural distribution such as *Lythrum salicaria* L. in Eurasia is likely to have broad variation in morphological traits and the potential for considerable phenotypic plasticity. This variation may be helpful for the species to adjust to environments outside of its native range and on other continents [2–3]. Few studies combine a comparison of genetic relationships to plant performance in multiple common garden environments along with corroborating field studies on different continents [4].

For ecologists, an important way to look at the relationship between performance and environment is to evaluate plant size in different climates. For example, at the colder range extreme of a species, plant size might be smaller because of reduced growing season and solar irradiance, and also at hotter extremes because of increased respiration and water loss [5]. Patterns of plant performance across geographical ranges or under varying common garden settings can give some insight into how well species might perform if temperatures change ([6, 1], respectively).

From the genetic perspective, a variety of mechanisms are used to explain the ability of invasive species to colonize outside of their native ranges (as reviewed in [7–8]). Even though the genetic diversity of founding invasive populations can be diminished because of bottlenecks [9], genetic diversity alternatively may be greater in such populations. For example, genetic admixture may increase genetic diversity when invasive propagules from diverse sources interbreed (as reviewed in [10–11]). Any enhanced genetic diversity in such populations may promote invasiveness by acting at the individual level through heterosis (i.e., heterozygote advantage [12]), or at the population level by producing a diverse array of phenotypes for selection to act upon [13]. These new environments also might be novel to the invasive species [14]. Thus, a genetic mixing event might generate general purpose genotypes capable of responding to a wide range of conditions with a high degree of plasticity (as reviewed in [15]). An invasive species with high plasticity might adjust quickly to novel conditions, with individuals more likely to spread in the population. Another consideration is that an invasive population may have lower competitive ability than native populations in its native ‘ancestral’ environment [16].

*Lythrum salicaria* has received attention from invasive species biologists because of its reported ability to supplant native species and alter community structure [16–22]. Evolutionary biologists have also been interested in this species’ noting that native and non-native populations are similar in their genetic diversity, at least partly because of population admixture [23–24]. Often, admixture leads to an increase in the size of individuals [24–29]. In addition, some studies suggest that *L*. *salicaria* has adapted to environmental differences along latitudinal gradients in North America by varying attributes such as height and flowering time [30–35] with shorter plants flowering earlier in the growing season in the North. For example, shorter non-native plants from northern environments flowered earlier than others, even when grown in a greenhouse [30]. In contrast, other studies suggest that trait variation in North America is due to random and not selective processes [24]. Apparently, populations of *L*. *salicaria* have very plastic responses to water and nutrient environments [28, 35]. Together, these findings paint a picture of *L*. *salicaria* as a genetically diverse, phenotypically plastic, and highly adaptive species. *Lythrum salicaria* has the capacity to tolerate a wide range of environmental conditions across broad latitudinal gradients through regional adaptation as well as locally through phenotypic plasticity as reflected in biomass differences of individuals from the same mother plants grown in different environments.

Global investigations of latitudinal differences in phenotypic response to environment are scant in *L*. *salicaria*, particularly studies that combine genetic, common garden and field observations Therefore, the objective of this study was to compare the phenotypic expression of native European and invasive North American populations of *L*. *salicaria* jointly established in two common gardens in a cold/dry vs. hot/humid environment (mean annual temperature, Czech Republic vs. Louisiana: 8.0° vs. 19.6°C, respectively; see Methods for further details of garden environment), as well as in field settings.

We tested the hypotheses that:

1. Overall patterns of total, stem/leaf, root and inflorescence biomass produced by individuals of *L*. *salicaria* differ depending on the latitude of seed origin regardless of whether grown in a cold/dry or hot/humid climate.
2. Genetic diversity of *L*. *salicaria* is similar in Eurasia and North America both within and among populations with respect to heterozygosity.
3. Phenotypic expression of inflorescence and total biomass is similar in gardens in Třeboň and Lafayette (cold/dry vs. hot/humid) as examined using reaction norms; thus, we would expect to find no evidence that phenotypic plasticity is affected by provenance.
4. Flowering time is earlier and plants are shorter among plants established from maternal plants from northern populations in both Eurasia and North America when grown in gardens in Třeboň and Lafayette (cold/dry vs. hot/humid) following [30].

## Materials and methods

### Study species and global habitats

*Lythrum salicaria* is a perennial wetland species of the Northern Hemisphere [36] with a broad native distribution in Eurasia including Europe [37], the Mediterranean, North Africa, northern and western Asia and the Himalayas [38]. *Lythrum salicaria* also has a limited occurrence in the Southern Hemisphere in south-east Australia, where the species may have become invasive [36]. The species invaded North America in the early 1800’s, and it now has a broad distribution in the northern part of the continent [22]. The northern limit in North America and Eurasia is about 51° and 65°N latitude, respectively, with limited occurrences farther northward [38]. Southward populations are sporadic, with occurrences in North Africa [38], and in North America and along the Mississippi River as far south as Venice, Louisiana (29.3°N latitude; David White, personal communication) and near Williams Pass of the West Bay Outfall, Louisiana (29.1°N latitude; Michael Massimi, personal communication). The species was listed in “Flora Americae Septentrionalis’ ([39] in [40]). *Lythrum salicaria* did not become invasive in North America until the 1930’s, but it is unlikely that the species became more invasive after it hybridized with *L*. *alatum* [23]. Nevertheless, repeated introductions by horticulturists created an opportunity for admixture (as reviewed in [10–11]). Worldwide, the species occurs in wet places such as wetlands, lake shores and disturbed ditches [36].

### Common garden design and morphological sampling

To assess any latitudinal differences in the biomass or flowering time of native vs. invasive populations of *L*. *salicaria*, we used germinated seedlings to establish common gardens in the cold/dry versus hot/humid climates of Czech Republic and Louisiana U.S.A. in April, 2006. The cold garden was established at the Institute of Botany, Academy of Sciences of the Czech Republic in Třeboň, Czech Republic (49°N, 14° 47’ E), and the hot garden at the U.S. Geological Survey, Wetland and Aquatic Research Center in Lafayette, Louisiana, USA (30°, 13’ N, 92°01’ W). Třeboň is characterized by a cooler, drier climate (mean annual temperature: 8.0°C; growing season length: 192 days; precipitation: 71 cm) than Lafayette (mean annual temperature: 19.6°C; growing season length: 228 days; precipitation: 149 cm). Using a hand-held thermometer in the hot common garden (Lafayette “garden” greenhouse), maximum and minimum temperatures during the growing season (May 27, 2008 –November 15, 2008) were compared to a weather station less than 1 mile from the garden (maximum temperature: 35.5° vs. 32.2°C; minimum temperature; 23.5° vs. 21.1°C; NOAA GHCND: USC00165021 [41]), so that conditions in the garden were somewhat warmer than those outside. To coordinate research methodology and reduce observer effects, some of the researchers from both continents worked in both gardens for periods of time throughout the study.

For the experiment, seeds from populations of *L*. *salicaria* were collected along two distributional gradients in Eurasia and North America (native vs. invasive, respectively) from four latitudes on each continent (geographical provenances of mother plants in Eurasia: Finland, Czech Republic, Spain, Turkey; in North America: Edmonton (Canada), Wisconsin, Illinois, Tennessee; Table 1). During collection, seeds from each of these eight populations were bagged separately from approximately twenty maternal plants, or genotypes, and each mother plant was given an identification number. Maternal plants were selected from three 1-m^2^ quadrats haphazardly located within *L*. *salicaria* patches within each population. The minimum distance between sampled individuals was 2 m and sampling was conducted only in *L*. *salicaria* patches exceeding 5 m in diameter.

**Table 1:** Seed collection locations of *Lythrum salicaria* used in the common garden studies in cold vs. hot gardens (Třeboň, Czech Republic vs. Lafayette, Louisiana).

Continent of seed origin	Geographic location	Place name	Latitude	Longitude	Maternal plants sampled	Seedlings sampled	Full-sib pairs	Seedlings analyzed	Markers scored
Eurasia	Finland	Vantaa River	60° 36’N	21° 26’ E	19	35	3	32	259
	Czech Republic	Branišov	48° 59’N	14° 24’ E	17	39	3	36	238
	Spain	Segre River	41° 37’N	0° 37’ E	12	18	0	18	200
	Turkey	Antalya	36° 52’N	31° 11’ E	7	7	0	7	200
North America	Edmonton	Wabamun Lake	53° 33’N	114°31’ W	15	24	1	23	238
	Wisconsin	Okee	43° 21’N	89° 34’ W	19	39	3	36	226
	Illinois	Arthur	39° 42’N	88° 28’ W	20	40	3	37	243
	Tennessee	Nickajack	35° 00’N	85° 37’ W	12	16	0	16	227
Totals					121	218	13	205	306

To create experimental subjects for our common gardens (Třeboň and Lafayette), we either sowed seeds from each numbered mother plant into plastic trays filled with sand and then transplanted three seedlings into pots (cold garden: Czech Republic), or we divided the seeds from each of the original mother plants into three equal portions and sowed them directly into 4 L pots (hot garden: Louisiana). Seeds from the same twenty mother plants were germinated in both common gardens and measured directly for performance. After true leaves were established in both common gardens (mid-May 2006), we retained one seedling pot^-1^ and removed the rest. The sand in each pot was kept moist but not flooded during the experiment. Each pot was fertilized using fertilizer (11:11:11 NPK) at a level of 7 g per liter of sand. In the cold garden, we placed the pots in outdoor tubs (120 x 180 x 50 cm^3^), while in the hot garden, we placed the pots in tubs in a greenhouse. In both gardens, we maintained the water level at half the height of the pots (water depth = 12 cm). This watering procedure assured continuous moist conditions without flooding. The hot garden was maintained under restrictions as outlined by the State of Louisiana and USDA (Permit # 37–86751) because of the invasive status of this species in North America.

One individual per mother plant was harvested each year (if available) at the end of the growing season in 2006, 2007 and 2008. Individuals were harvested after flowering had reached halfway up the main inflorescence, which assured near-maximum plant development without great loss of leaves, and that the individuals were harvested at the same phenological stage. Note that growth stops after flowering in this species [29]. Because of differences in the flowering phenology, harvesting began in early July and continued until early October in the cold common garden, and continued from late August through late September in the hot garden. We recorded the date of harvest for each individual. The number of individuals harvested in each garden differed among the populations because of mortality during the growing season, but typically 15 or 16 individuals per population (representing yearly median values) were harvested. Only the population from Spain experienced mortality sufficient to reduce yearly sample sizes below ten individuals.

We measured all harvested individuals for vegetative performance (above-ground (stems/leaves), below-ground (roots), and total biomass), and reproductive performance (inflorescence biomass). Immediately upon removal of individuals from pots, we carefully washed the sand from the roots and rootstock, and separated the inflorescences from stems and leaves, placed them into individual bags, and dried the material in a drying oven at 70°C for 72 hours. Tissue components were weighed separately for each individual (i.e., above-ground stems and leaves, below-ground roots, and inflorescences).

In the field, heights of *L*. *salicaria* individuals were measured by volunteers in the Purple Loosestrife Volunteer program in Eurasia, Asia, North America and Australia [42] (S1 Table). Measurements were made after flowering occurred and plants had reached their maximum height. Volunteers selected three field individuals to measure by randomly tossing a rock in a wetland, placing a 1 m^2^ quadrat, and then measuring the tallest individual in the quadrat [42].

### Molecular protocols

To assess genetic diversity differences between Eurasian and North American populations of *L*. *salicaria*, we genotyped a subset of culled seedlings from early in our experiment (see *Experimental Design and Morphological Sampling*, above). We randomly chose three healthy individuals per maternal plant, which were held at -20°C prior to DNA extraction. We extracted total genomic DNA from leaf tissue with the Invisorb Spin Plant Mini Kit (Invitek, Germany) following the manufacturer’s protocol with the following modifications. Plant material was homogenized directly in lysis buffer with Proteinase K and incubated in a shaker for 30 minutes at 65°C. The homogenate was then centrifuged at 14,000 rpm for 5 minutes, and the liquid portion transferred onto the pre-filter provided. This additional step aided in the removal of secondary metabolites, which often caused plugging of the pre-filter. DNA was eluted in 50 μl of the elution buffer provided.

We assessed population-level genetic diversity using amplified fragment length polymorphisms (AFLPs), following procedures similar to those of [43], as outlined in [44–45]. For the pre-amplification reaction, we used adenine as the selective nucleotide in the primers matching both the *Eco*RI and *Mse*I restriction sites. For the subsequent selective restriction fragment amplification reactions, we used four primer combinations, each with *Eco*RI-ACG as the primer matching the *Eco*RI-site, and with *Mse*I-AGT, *Mse*I-AAC, *Mse*I-AAG, and *Mse*I-ACA as the primers matching the *Mse*I-site. We resolved amplified fragments on an ABI PRISM 310 Genetic Analyzer in the presence of an internal size standard (GeneScan-400HD ROX; Applied Biosystems, Inc., Foster City, California, USA). We scored genetic profiles for the presence or absence of a select set of markers using Genographer, Version 1.6 [46], following the methods of [45]. Marker reproducibility was confirmed by running replicate reactions on a subset of our samples.

### Statistical analyses

Plant tissue components. For the common garden study, total biomass was calculated by summing the dry biomasses of each tissue component (above-ground, below-ground and inflorescence biomass) for each individual. Biomass is reported throughout the paper as biomass pot^-1^, noting that each pot had only one individual. To select the best model fit to compare the total biomass allocated to various plant tissue components (above-ground, below-ground and inflorescence biomass), we used multiple regression with tissue type as a categorical variable and class effects of common garden type (cold/dry vs. hot/humid: Třeboň Czech Republic vs. Lafayette LA USA, respectively), geographical provenance of mother plant (Table 1), year of study (2006–2008), and their interactions in the General Linear Model framework of Proc GLMSELECT [47] including linear, log, quadratic and non-parametric index transformations of response data to choose best fit equations. An index is a composite statistic that looks at representative data points to summarize and rank observations. We used the index to introduce a ranked non-parametric variable into our statistical model. Here, the approach is useful, because the pattern of the response variables (biomass and heterozygosity) might vary geographically. This approach allowed us to tease out how *Lythrum* responses may change across geographical ranges in Eurasia versus North America. Thus, the GLM Select procedure fit a combined equation of linear, log linear, quadratic and index components to describe the non-linear behavior of the response in various parts of the latitudinal range. Informative variables were identified with Stepwise Regression (forward and backward) with p-value thresholds of more than 0.15 not entered into the ANOVA model. Continent of seed origin (as related to the geographical provenance of mother plant) was one of the variables that was deleted from further analysis in this tissue analysis procedure (p > 0.15). Following the tests, multiple comparisons were made using p-values adjusted for the Bonferroni experiment-wise error rate. Appropriate transformations were made to meet the assumptions of ANOVA [48]. Using the same analysis approach, field heights of *L*. *salicaria* were analyzed with the General Linear Model framework using continent, latitude and their interactions. Note that covariance estimates show that the responses of mother plants within a latitude had no correlation under normality of the residuals of the model (independence). For all models run, normality and homogeneity were tested at alpha = 0.05, SAS 9.4 2002–2012) (p < 0.0001; SAS 9.4 2002–2012). Therefore, these data are not pseudo-replicated under the normality assumption (Montgomery 1991).

Using a Principal Components Analysis approach, responses of plants grown from seeds of the same maternal plants were compared in the Třeboň vs. Louisiana gardens in 2006–2008. The cross-products matrix was estimated using correlation coefficients while parameter scores were calculated as distance-based biplots [49]. Monte Carlo randomization tests were conducted (999 runs) to determine significant axes for the Principal Components Analysis using PC Ord Version 6 [49]. For the datasets from 2006, 2007 and 2008, the first three axes were significant in the PCAs (p < 0.05), while only axes 1 and 2 were significant (p < 0.05) for the combined dataset of all years (2006–2008). Pearson correlation coefficients determined variables that were most related to each axis (r^2^ > 0.4).

#### Reaction Norms

Reaction norms were developed from inflorescence and total biomass of plants grown in Třeboň vs. Lafayette from 2006–2008 to further explore differences in phenotypic expression of plants grown from seeds of the same maternal plants in different environments. Note that genotypes at the maternal level showed no differences within populations (p > 0.05). Reaction norm responses were compared using population and continent as class variables in ANOVA. Trait differentiation (Q_ST_) was computed for log above-ground, below-ground, inflorescence and total weight using ANOVA with population as a class variable (SAS 2012).

#### Genetic data. Maternal sibship

Before examining genetic diversity within and among populations, we analyzed our known maternal sibships for the presence of full siblings using the program COLONY [50–51]. Full siblings are particularly problematic for inferring population-level genetic parameters based on simulation modeling because of genetic redundancy [52]. Thus, we ran a single COLONY analysis separately for each of our collection sites selecting “without inbreeding” and both “male polygamy” and "female polygamy" in the program, because *L*. *salicaria* is an outcrossing, monoecious species due to its heterostylous habit [38], with polygamy on the part of both males and females. Known sibships were identified prior to each medium-length run, which employed the full-likelihood method at medium precision with no sibship prior, and treated population allele frequencies as unknown. We omitted from further analysis all but one individual from each full-sib dyad (pair of full siblings) in each instance where COLONY assigned a > 0.05 probability of a full sibship. Individuals with a high number of missing (i.e., unscorable) AFLP markers were omitted. In cases where the number of missing markers was equal between the two members of a full-sib dyad, one member was chosen randomly.

#### Population-level diversity

We compared genetic diversity between *L*. *salicaria* grown from seeds from Eurasian and North American populations using population-level diversity indices developed for use with dominant markers [53]. We report the number of polymorphic loci (i.e., markers present in 5 to 95% of samples), and Nei’s gene diversity (i.e., expected heterozygosity), calculated using the software program AFLP-SURV [54]. For our calculations of gene diversity, allele frequency estimates were derived using the Bayesian approach of [55] with non-uniform priors, assuming Hardy-Weinberg genotypic proportions. We also report polymorphism information content, or PIC [56] as a measure of genetic diversity, which is calculated as 2*f*(1-*f*) averaged over AFLP markers, where *f* is the proportion of individuals with the marker present.

#### Heterozygosity

We calculated a measure of individual heterozygosity for each individual to determine the extent to which it was affected by common garden type, continent of seed origin, latitude of seed collection (Table 1), and their interactions. Individual heterozygosity was calculated according to [57] as: $I\;=\;\sum_{i\;=\;1}^{n}q_{i}x_{i}/\sum_{i\;=\;1}^{n}q_{i}$, where *q*_*i*_ is the frequency of the recessive (null) AFLP allele at each locus and *x*_*i*_ is 1 for marker presence and 0 for marker absence.

*Population genetic structure*. We determined if genetic structure of populations was present across latitudinal gradients by examining population differentiation in Eurasia vs. North America. Our initial approach was to use the Bayesian clustering algorithm [58] in STRUCTURE, Version 2.3., to determine if population structure was related to location. This algorithm searches for *K* clusters of individuals adhering to Hardy Weinberg equilibrium frequencies and linkage equilibria among marker loci, with prior values of *K* defined by the user. We used the RECESSIVEALLELES option provided in STRUCTURE, which compensates for the ambiguity inherent in dominant markers such as AFLPs. We compared posterior probabilities of *K* = 1–8 clusters using the *ad hoc* statistic, Δ*K* [59], because the true *K* of the homogeneous dispersal of individuals between populations could not be assumed. To assure an adequate burn-in length to achieve chain convergence, an initial burn-in length of 1 X 10^5^ followed by 1 X 10^6^ MCMC repetitions was run for each value of K and the consistency of these results to all subsequent results was confirmed. Subsequent runs (20 independent runs per *K*) used a burn-in length of 3 X 10^4^, followed by 1 X 10^5^ MCMC repetitions. Note that we ruled out the role of selection in inflating apparent differentiation among our populations due to the inclusion of a limited number of non-neutral AFLP markers, by conducting a genome scan for such markers using BayeScan version 2.1 (at a q-value threshold of 0.05). We then re-ran our STRUCTURE analysis without these markers, of which there were just 5 (of 311 total), and found there to be no change in our results. Thus, these results are not presented. All models assumed admixture and correlated allele frequencies.

## Results

### Tissue allocation in cold vs. hot garden environments as a function of latitude and continent of origin

**Fig 1:**
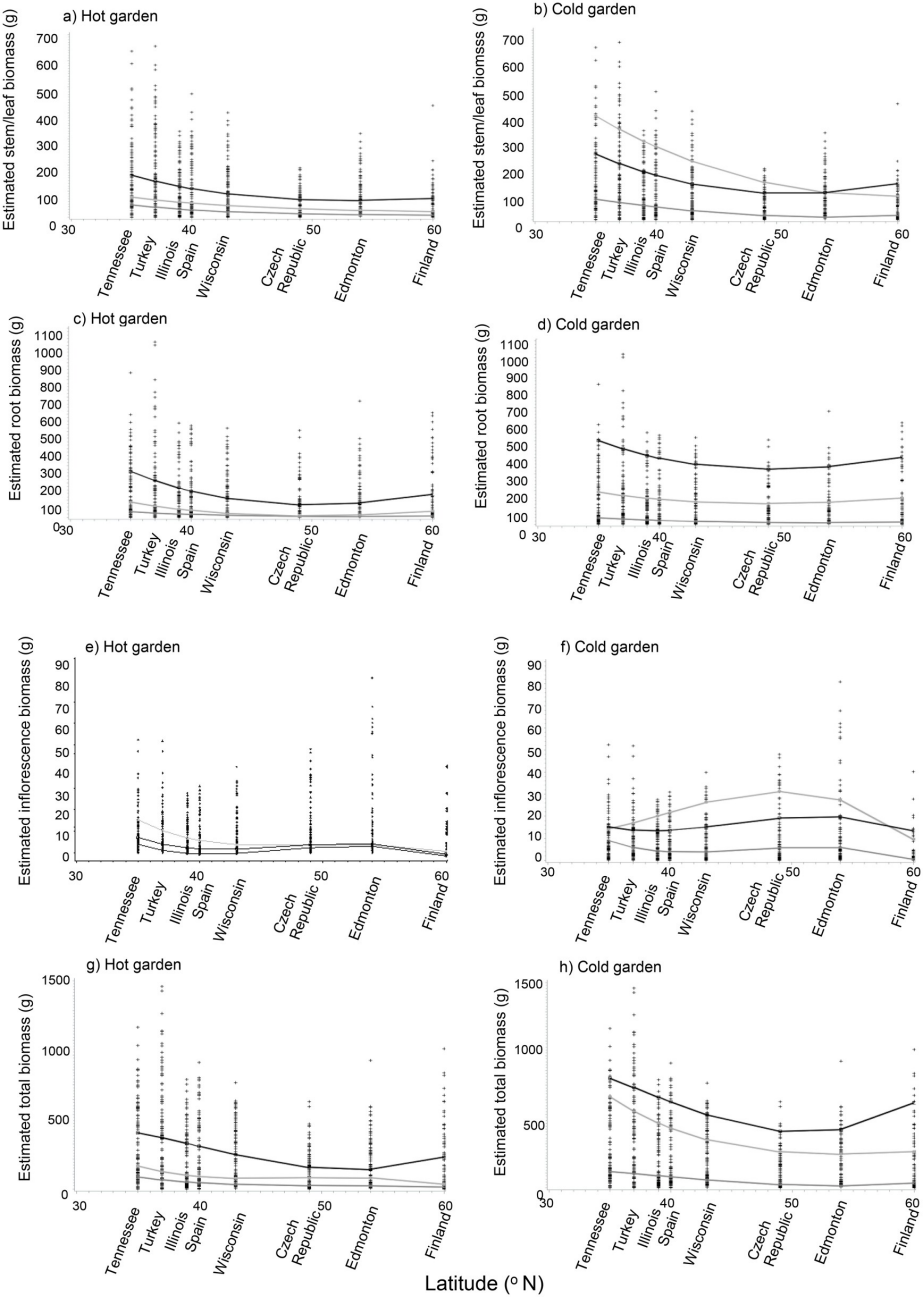
Oven-dried biomass (g) of various tissue types of *Lythrum salicaria* in two experimental gardens in Třeboň, Czech Republic vs. Lafayette, Louisiana (cold/dry vs. hot/humid garden, respectively) including(a-b) above-ground stem/leaf, (c-d) below-ground root, (e-f) above-ground inflorescence and (g-h) total above and below-ground biomass pot^-1^ (g) versus latitude of seed origin during 2006, 2007, and 2008 (light gray, gray and black lines, respectively). Populations from seeds collected on the same continent as the common garden are designated in bold font. See Table 1 for the ANOVA table.

None of the biomass variables (above-ground, below-ground, inflorescence, total biomass) showed significant phenotypic differences among mother plants within garden types (Lafayette vs. Třeboň: hot vs. cold, respectively), latitudes of seed origin, or years of study (Table 2A–2D; p > 0.05). Above-ground biomass generally increased in every year of the study (Table 2A), and was higher in the Třeboň than the Lafayette garden except for the first year of the study (Fig 1B and 1A; Bonferroni: p = 0.1571; S1, S2 & S3 Figs). Above-ground biomass did not differ in the Třeboň garden between 2007 and 2008 in individuals originating from seed collected from higher latitudes (Table 2A; Fig 1B; Bonferroni: p = 0.2212). Similarly, below-ground biomass increased in every year of the study among individuals from all latitudes (p <0.0001; Table 2B; Fig 1C and 1D), but with no differences between the Lafayette vs. Třeboň garden from 2006–2008 (p > 0.05). Total biomass generally had lower values in individuals grown from seeds collected from the middle of the range on both continents (i.e., U-shaped distribution; Table 2D; Fig 1G and 1H; p = 0.0466 to < 0.0001) after the first year of the study (p = 0.1571). Total biomass was higher in individuals grown in Třeboň than Lafayette after the first year of the study (p = 0.1571; Fig 1G and 1H and S1, S2 & S3 Figs). By 2008, the plants grown in Třeboň had higher values for several biomass variables (e.g., above-ground biomass, root biomass; AboveDW, RootDW, respectively), whereas the shoot/root ratio was higher in the Louisiana garden (S3 Fig).

**Table 2:** ANOVA table of the effects of sources of variation on tissue biomass (g) of *Lythrum salicaria* including total (a), above-ground (b), below-ground (c) and inflorescence (d).

Sources of Variation	df	MS	F	p		Means ± S.E.
**(a) Stem/leaf biomass (r**^**2**^ **= 0.7133)**						
Model^1^	19	344167.2	104.4	<0.0001	***	
Error	797	3298.0				
Total	816					
Garden x year	5	901615.1	273.4	<0.0001	***	
Garden x latitude x year	6	291421.2	88.4	<0.0001	***	
Garden x latitude x latitude	2	118027.8	35.8	<0.0001	***	
Garden x Ilatitude x year	6	7753.0	2.4	0.0295	*	
**(b) Root biomass** (r^2^ = 0.7657)						
Model	11	1377738.7	239.1	<0.0001	***	
Error	805	5761.9				
Total	816					
Year	2	4830775.3	838.4	<0.0001	***	
	2006					12.9 ± 0.9^a^
	2007					81.9 ± 4.5^b^
	2008					278.2 ± 11.5^c^
Latitude x year	3	139062.4	24.1	<0.0001		
Garden x Ilatitude x year	6	846064.7	146.8	<0.0001		
**(c) Inflorescence biomass** (r^2^ = 0.5370)						
Model	18	3015.7	51.4	<0.0001	***	
Error	798	58.7				
Total	816					
Garden	1	22409.7	382.1	<0.0001	***	
Cold (Třeboň)						177.2 ± 9.1^a^
Hot (Lafayette)						3.5 ± 0.3^b^
Year	2	1707.6	29.1	<0.0001	***	
2006						5.7 ± 0.4^a^
2007						10.3 ± 0.8^b^
2008						9.8 ± 0.6^c^
Garden x year	2	8479.2	144.6	<0.0001	***	
Latitude	1	560.1	9.6	0.0021	**	
Latitude x year	2	664.4	11.3	<0.0001	***	
Garden x latitude x year	3	1150.3	19.6	<0.0001	***	
Garden x latitude x latitude x year	6	596.8	10.2	<0.0001	***	
ILatitude	1	2577.9	44.0	<0.0001	***	
**(d) Total biomass (r**^**2**^ **= 0.7600)**						
Model	21	1683634.2	120.2	<0.0001	***	
Error	797	14002.1				
Total	818					
Garden x latitude x latitude	2	4847113.5	346.2	<0.0001	***	
Latitude x latitude x year`	2	6447829.7	460.5	<0.0001	***	
Garden x year	5	2012873.0	143.8	<0.0001	***	
Garden x latitude x year	6	411443.8	29.4	<0.0001	***	
Garden x ILatitude x year	6	38900.5	2.8	0.0111	*	

“I” signifies the index of the variable. Only significant main effects and interactions are reported in this Generalized Linear Model approach. Actual means ± S.E. are given for significant non-interacting main effects; patterns of significant interactions are shown in figures. Significance is indicated by ‘***’, '**' and ‘*” (p ≤ 0.001, p ≤ 0.01, p ≤ 0.05, respectively). For multiple comparisons of means, significant differences are indicated with different letters based on a Tukey-Kramer test.

^1^ Note that the equation for the best fit model relationship of total biomass to tissue components, latitude of seed collection and common garden location was: total biomass = 6.446139154—0.154182726 * latitude + 0.816450304 * below-ground biomass*log(year = 2006) + 0.003045939 * below-ground biomass* below-ground biomass* I(year = 2006) + 1.083280923* below-ground biomass* I(year = 2007) + 0.000471497 * below-ground biomass* below-ground biomass*I(year = 2007) + 1.265310141 * below-ground biomass* below-ground biomass* I(year = 2008) + 0.000043426 * below-ground biomass* below-ground biomass* I(year = 2008)—0.343412573 * below-ground biomass* below-ground biomass* I(common garden = cold) + 4.341390304 * log below-ground biomass* I(common garden = cold)—0.3224302 * log below-ground biomass* I(common garden = hot) + 1.033745873 * above-ground biomass* I(common garden = cold) + 0.894323868 * above-ground biomass*I(common garden = hot)—2.528515029 * log inflorescence biomass* I(common garden = cold) + 4.163739227 * log inflorescence biomass* I(common garden = hot).

Inflorescence biomass was higher in Třeboň than Lafayette (14.1 ± 0.6 vs. 3.5 ± 0.3 g mean ± S.E. inflorescence) during the study (p < 0.0009), except during the first year (p = 0.1236; Table 2C; Fig 1E and 1F). Inflorescence biomass varied in a non-linear fashion as a function of latitude, keeping everything else constant (ILatitude: F = 44.0, p < 0.0001). In 2008, the plants grown in Louisiana took longer to flower than in Třeboň (GrowTime; S4 Table), although this variable was not significant in the PROC GLM Select analysis (p > 0.15).

Total biomass varied in a non-linear fashion as a function of garden type, latitude of seed collection and year (F = 2.8, p = 0.0111; Table 2D). Also note that the plants were not likely to have been pot-bound at the end of the study because the root biomass increased by 3.4 x between the 2^nd^ and the 3^rd^ year (mean root biomass in 2006, 2007 and 2008: 12.9 ± 0.9 g, 81.9 ± 4.5 g, and 278.2 ± 11.5 g pot^-1^, respectively).

**Fig 2:**
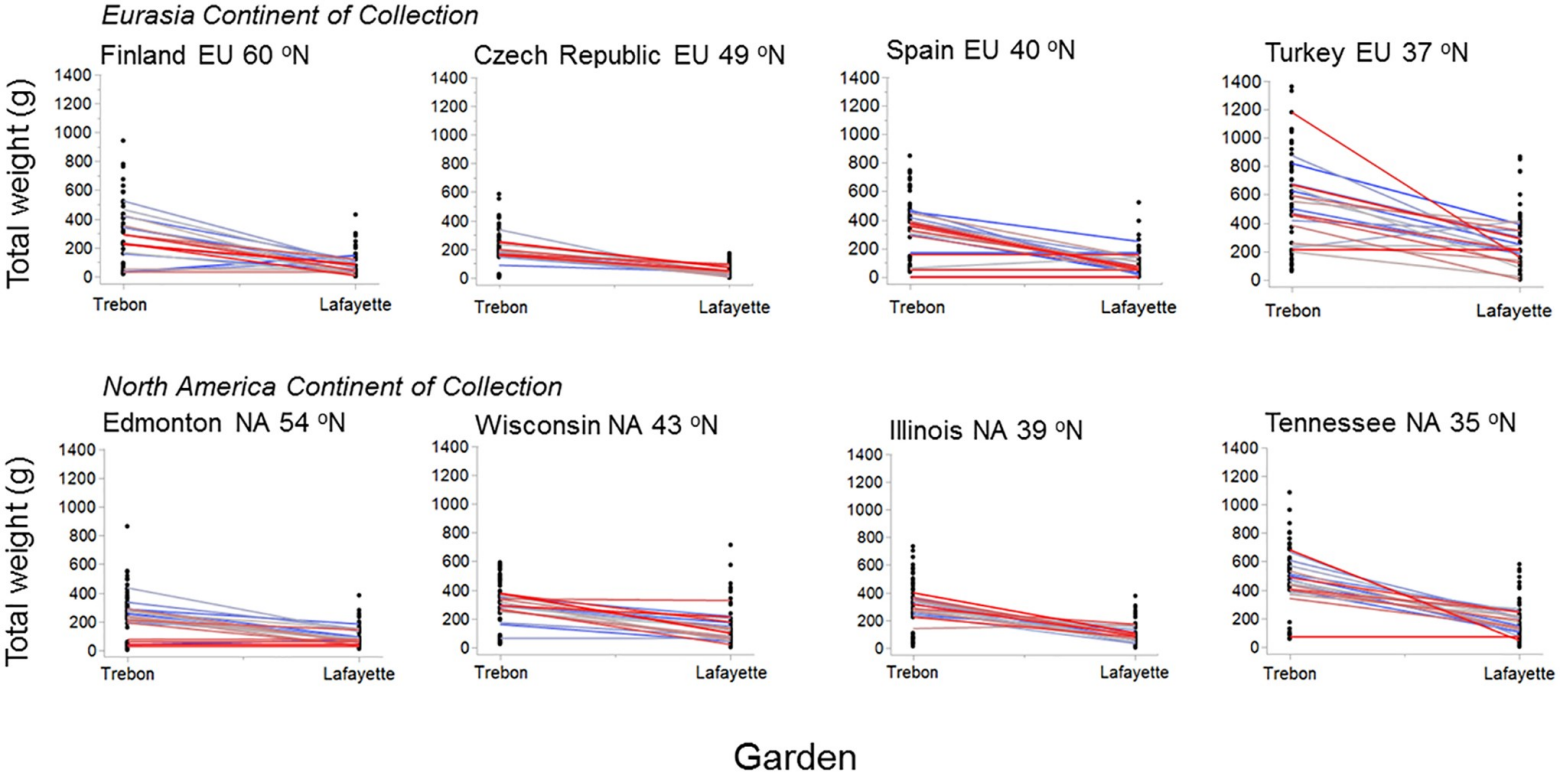
Reaction norms for total biomass (g) constructed from plants grown from the seeds of the same maternal plant grown in the gardens in Třeboň, Czech Republic and Lafayette, Louisiana (cold/dry vs. hot/humid garden). Individual maternal plants are depicted by a gradient of blue to red lines.

**Fig 3:**
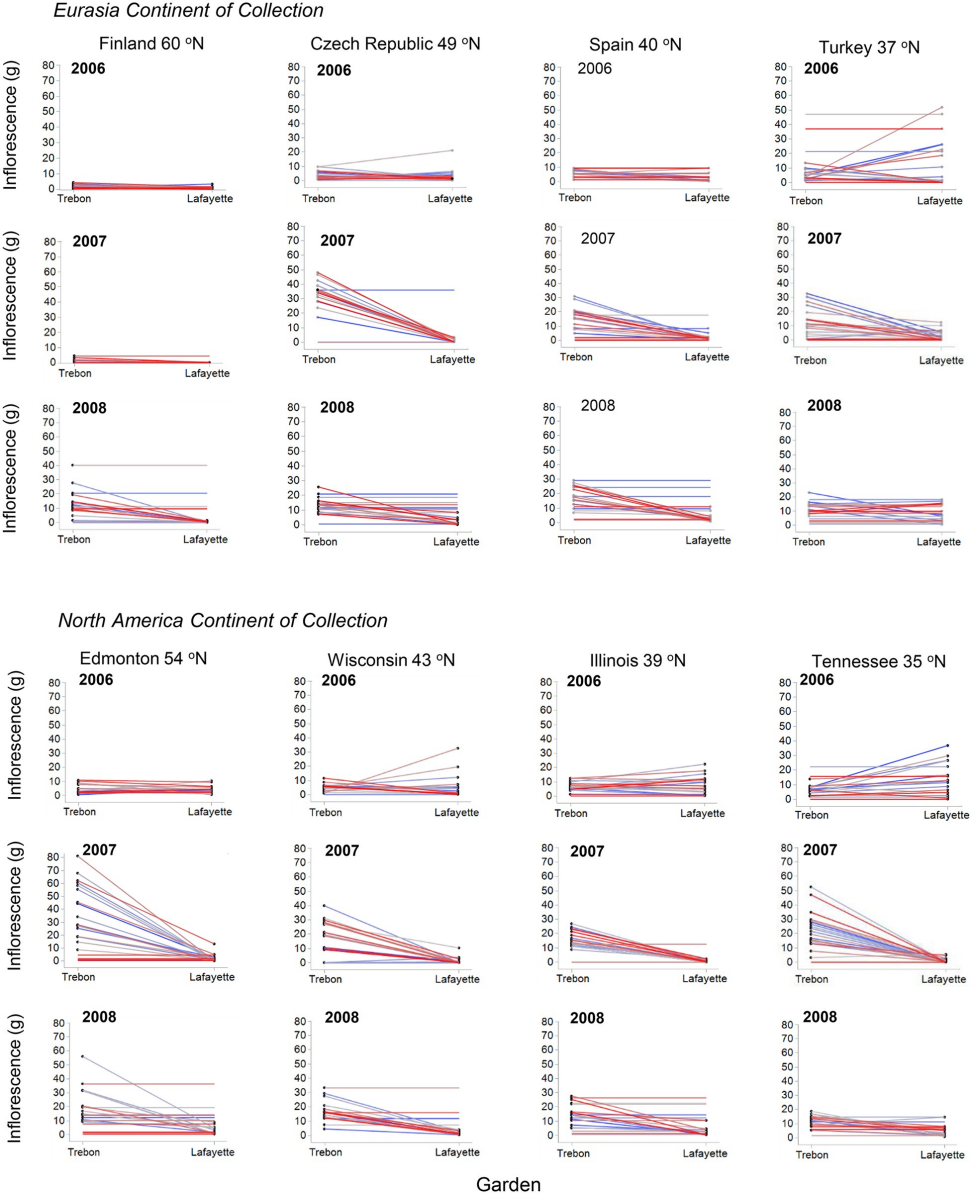
Reaction norms for log inflorescence biomass (g) constructed from plants grown from the seeds of the same maternal plant grown in the gardens in Třeboň Czech Republic and Lafayette Louisiana (cold vs. hot garden) in 2006–2008. Individual maternal plant numbers are depicted by a gradient of blue to red lines.

Reaction norms were constructed using progeny from the same maternal plants [60] compared between common garden environments in Třeboň vs. Lafayette (cold vs. hot garden; Figs 2 and 3). Note that GLM Select did not find significant differences in overall response among mother plants within populations (Table 2A–2D; p > 0.05). Broadly comparing log total biomass, individuals grown from the seeds of mother plants from Turkey, Spain and Tennessee had a wide variation in response when grown in Třeboň vs. Lafayette (F = 6.0, 2.5 and 3.4, respectively; p < 0.0001, p = 0.0120 and p = 0.0008, respectively; Fig 3). In general, plants grown in Lafayette had lower total biomass than their maternal counterparts when grown in the Třeboň garden (F = 12.0, p < 0.0001; Fig 2). The overall model of the reaction norms for log inflorescence weight was significantly different for maternal plants grown in the cold/dry vs. hot/humid garden (F = 10.9, p < 0.0001; Fig 3), with log inflorescence weight for populations significantly higher when grown in the cold/dry vs. hot/humid garden for populations in 2006 from Turkey, Spain, Czech Republic and Tennessee (F = 5.1, 3.4, 2.3, 3.2, respectively; p < 0.0001, p = 0.0007, p = 0.0199 and p = 0.0017, respectively); in 2007 from Turkey, Spain and Czech Republic (F = 4.3, 3.8, 5.1, respectively; p < 0.0001, p = 0.0002 and p < 0.0001, respectively); and, in 2008 from Turkey, Spain and Czech Republic (F = 2.8, 2.7, 2,0, respectively; p = 0.0050, p = 0.0077 and p = 0.0463, respectively). The continent effect comparing the reaction norm slopes in Eurasia vs. North America show that Eurasia had the larger values for reaction norms for above-ground and total biomass (Figs 2 and 3). Note that Q_ST_ and reaction norm for log total biomass were both significant, whereas for log inflorescence, reaction norms were significant but Q_ST_, using a Q_ST_ cut-off value of 0.2, was not (Q_ST_ log total biomass vs. inflorescence biomass: 0.117 vs. 0.049, respectively). The QST values for above-ground, below-ground, inflorescence and total biomass were as follows: 0.044, 0.044, 0.049 and 0.117, respective; p < 0.0001).

### Height differences along the latitudinal gradient in Eurasia vs. North America

**Fig 4:**
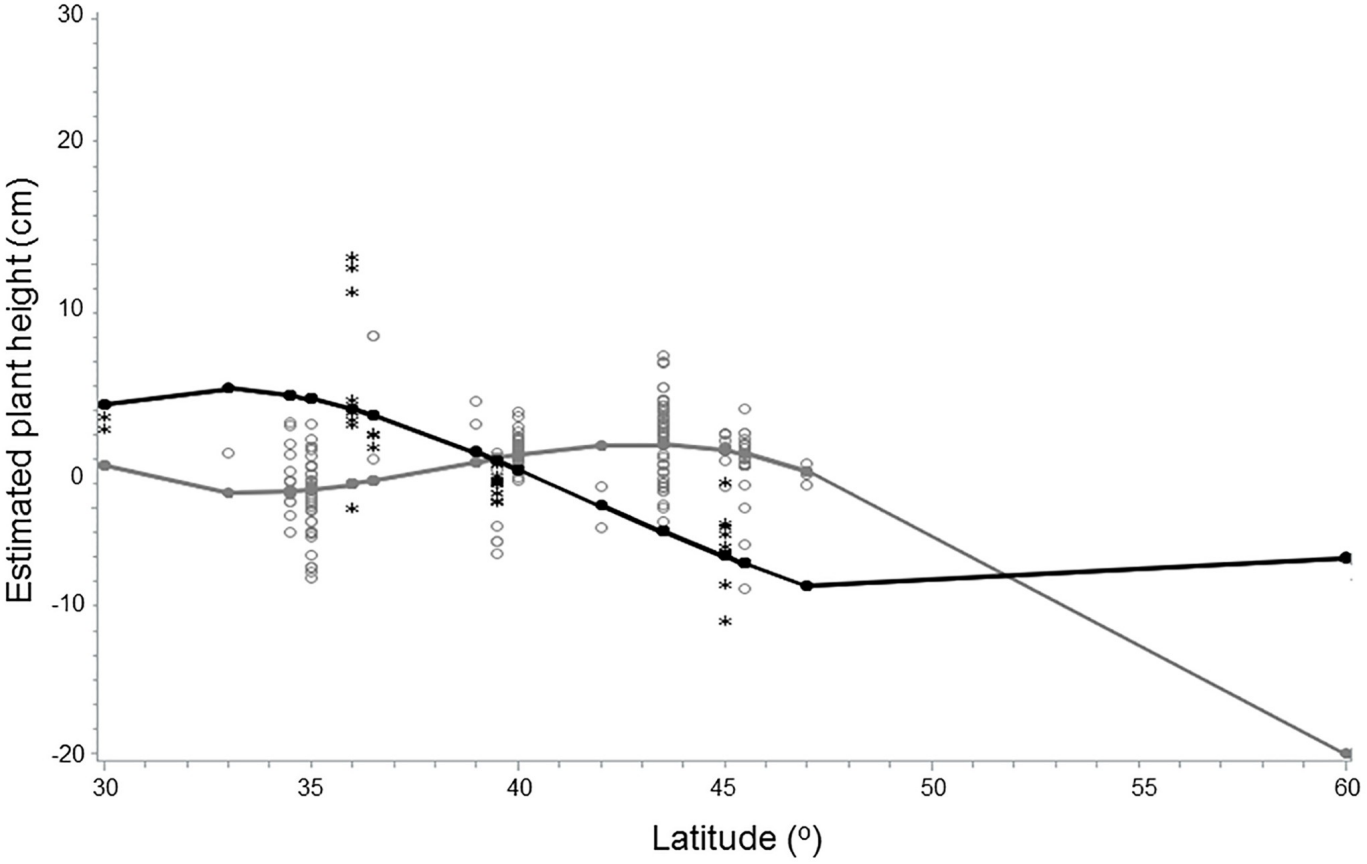
*Lythrum salicaria* heights (cm) in wetlands of Eurasia vs. North America as measured by volunteers in the Purple Loosestrife Volunteer Network (r^2^ = 0.361, p < 0.0001). The interpolated lines represent estimated plant heights for Eurasia and North America (filled black circle/line vs. gray circle/line, respectively). Means of measured heights latitude^-1^ are represented by stars and triangles (Eurasia and North America, respectively).

In Eurasia and North America, maximum individual heights (cm) were measured by volunteers at Kursuhlu Waterfall in Turkey and Samburg Tennessee USA (~36° and 36.5°N latitude, respectively; 413 and 291 cm, respectively; S1 Fig). In the southern part of the ranges, minimum heights were measured in Karakuya Lake, Turkey and Gurley, Alabama (36° and 34.5°N; 100 and 81 cm, respectively). In the North, the shortest plants were near Vantqaa-Tikkurila Finland, and Amos Canada (48° and 60°N latitude, respectively; 80 and 60 cm, respectively). Height decreased from the southern to the northern part of the range in Eurasia (Fig 4). Height patterns differed along the latitudinal gradients in both Eurasia and North America i.e. a continent x latitude interaction; plants were taller in Eurasia than in North America moving from south to north with the pattern switching northward of 40°N latitude (linear: t = -3.39, p = 0.0008; 2^nd^ order polynomial: 3.9, p < 0.0001; log linear: t = 4.7, p < 0.0001; Table 3; Fig 4). Best fit model relationship of the square root height to latitude was for Eurasia = 1776.109–2976.208 + latitude * (41.051–66.805) + latitude^2^ * (-0.265 + 0.414) + log latitude * 539.699; and for North America = 1776.109 + latitude * 41.050537 + latitude^2^ * (-0.265) + log latitude * (-802.945).

**Table 3:** ANOVA table of the effects of sources of variation on plant height (cm) of *Lythrum salicaria*, as measured by volunteers as part of a worldwide program.

Sources of Variation	df	MS	F	p	
**Height per plant (cm)**					
Model	7	58.5	25.9	<0.0001	***
Continent	1	26.0	11.5	0.0008	**
Continent x latitude	1	31.2	13.8	0.0002	**
Continent x latitude^2^	1	33.8	15.0	0.0001	
Continent x log latitude	2	30.3	13.4	<0.0001	***
Error	320	2.3			
Total	327				

Only significant main effects and interactions are reported in this Generalized Linear Model approach. Significance is indicated by ‘***’ and '**' (p ≤ 0.001, p ≤ 0.01, respectively).

### Genetic differences and population structure along the continental gradient

Among the 218 samples representing 8 total populations that we initially genotyped, COLONY revealed a total of 13 full-sib dyads. After the removal of one member of each pair, the total number of samples entering our analysis of genetic diversity was 205, which were distinguished on the basis of 306 AFLP markers generated from four primer pairs. The number of scorable markers ranged from 200 to 259 within populations (mean of 229 ± 21 SD), with the proportion of polymorphic markers ranging from 56 to 65%.

**Fig 5:**
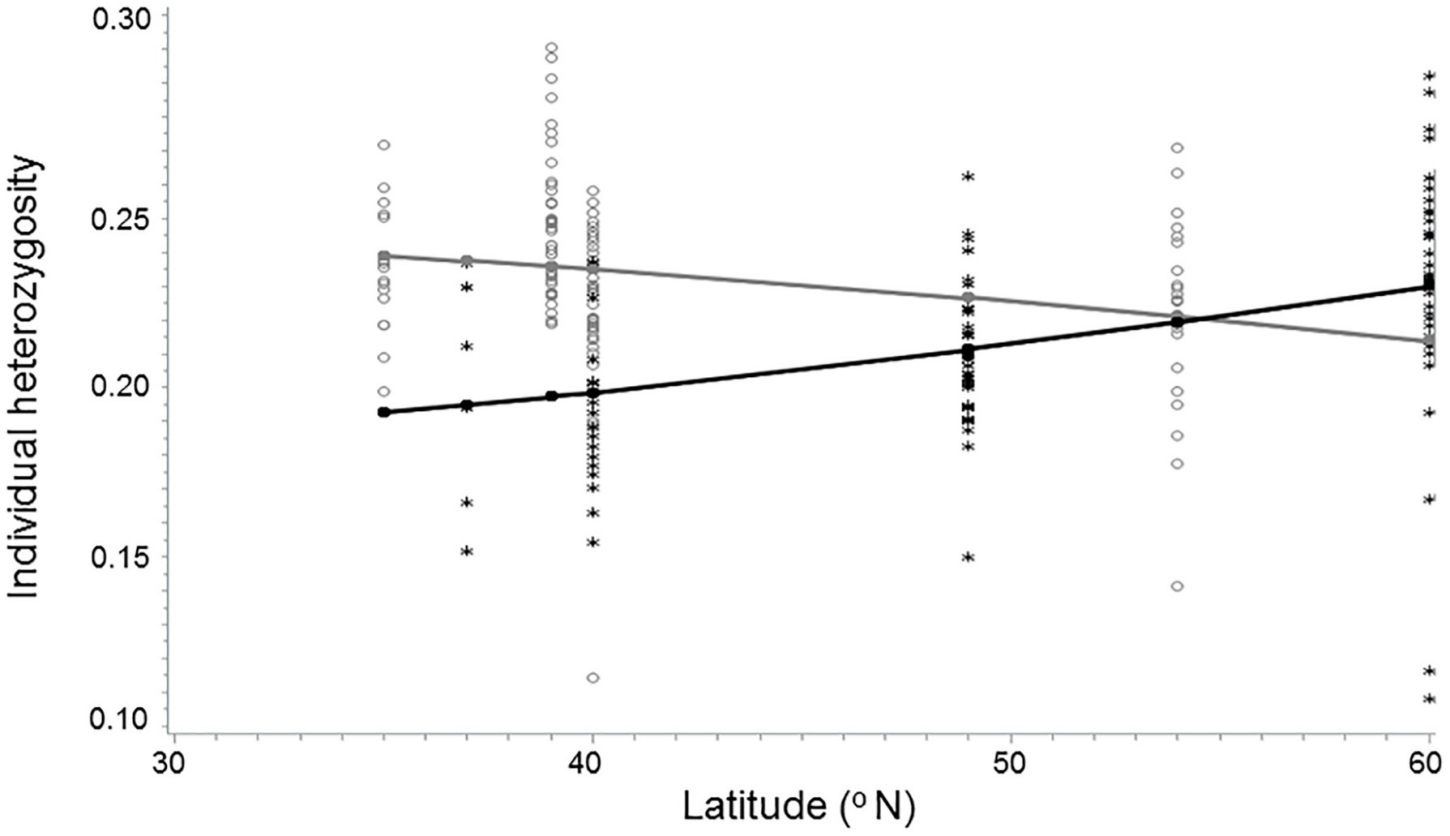
Individual heterozygosity for *Lythrum salicaria* individuals germinated from seeds collected along latitudinal gradients in Eurasia and North America (filled black circle/line vs. gray circle/line, respectively). Lines were fitted incorporating linear, second order polynomial and log linear equation components in PROC GLMSelect. Interpolated lines follow predicted values for Eurasia and North America (“*” vs. “*”, respectively).

Individual heterozygosity within populations increased curvilinearly northward in Eurasia but decreased northward in North America (i.e., continent x latitude^2^ interaction: t = 4.63 and -2.40, respectively; p < 0.0001 and 0.0172, respectively; Table 4; Fig 5). Thus, mean heterozygosity of seeds in Eurasia was highest in the north and lowest in the south (Finland vs. Turkey; 60° vs. 36°N latitude, respectively; Fig 5). In North America, the heterozygosity was highest in the south and lowest in the north (Chattanooga Tennessee USA vs. Edmonton Canada; 35° and 53°N latitude, respectively; Fig 5).

**Table 4:** ANOVA table of the effect of sources of variation on individual heterozygosity of *Lythrum salicaria* in Eurasia versus North America.

Sources of Variation	df	MS	F	p	
**Heterozygosity**					
Model	3	<0.01	10.5	<0.0001	***
Continent	1	0.01	9.0	0.0033	**
Continent x latitude^2^	2	0.01	10.6	<0.0001	***
Error	105	<0.1			
Total	108				

Significance is indicated by ‘***’ and '**' (p ≤ 0.001, p ≤ 0.01, respectively).

Note that equations for the best fit model relationship for heterozygosity were fitted incorporating linear, second order polynomial, log linear, and ranked index components into the equations: for Eurasia: 0.3041761445 –latitude * 0016735605**;** and for North America: 0.3041761445–0.1670273817+ latitude * 0.0015821897; r^2^ = 22.3. The overall model, continent and the interaction of latitude x continent were significant (p<0.0001, <0.0001, and 0.0033, respectively). Linear regression slope estimates for Eurasia and North America were significant (t = 3.41 and -3.09; p < 0.0009 and 0.0026, respectively).

We also compared population-level genetic diversity among *L*. *salicaria* populations. For these analyses, the ranges of similarities for the populations in Eurasia vs. North America included the proportion of polymorphic markers (56.2%– 65.4% vs. 55.9%– 65.0%, respectively), Nei’s gene diversity (0.168–0.218 vs. 0.187–0.211), and polymorphism information content (0.130–0.170 vs. 0.148–0.185, respectively) (S4 Table). These genetic diversity indices did not show any differences between continent, latitude, or the interaction of continent and latitude (p > 0.05; S4 Table).

Time to flowering (GrowTime) was longer in Lafayette than in Třeboň (S3 Fig) although not statistically significant in the GLM Select analysis (p > 0.05). We did not find that the flowering time differed in a predictable way based on the geographic provenance of mother plants (p > 0.05).

**Fig 6:**
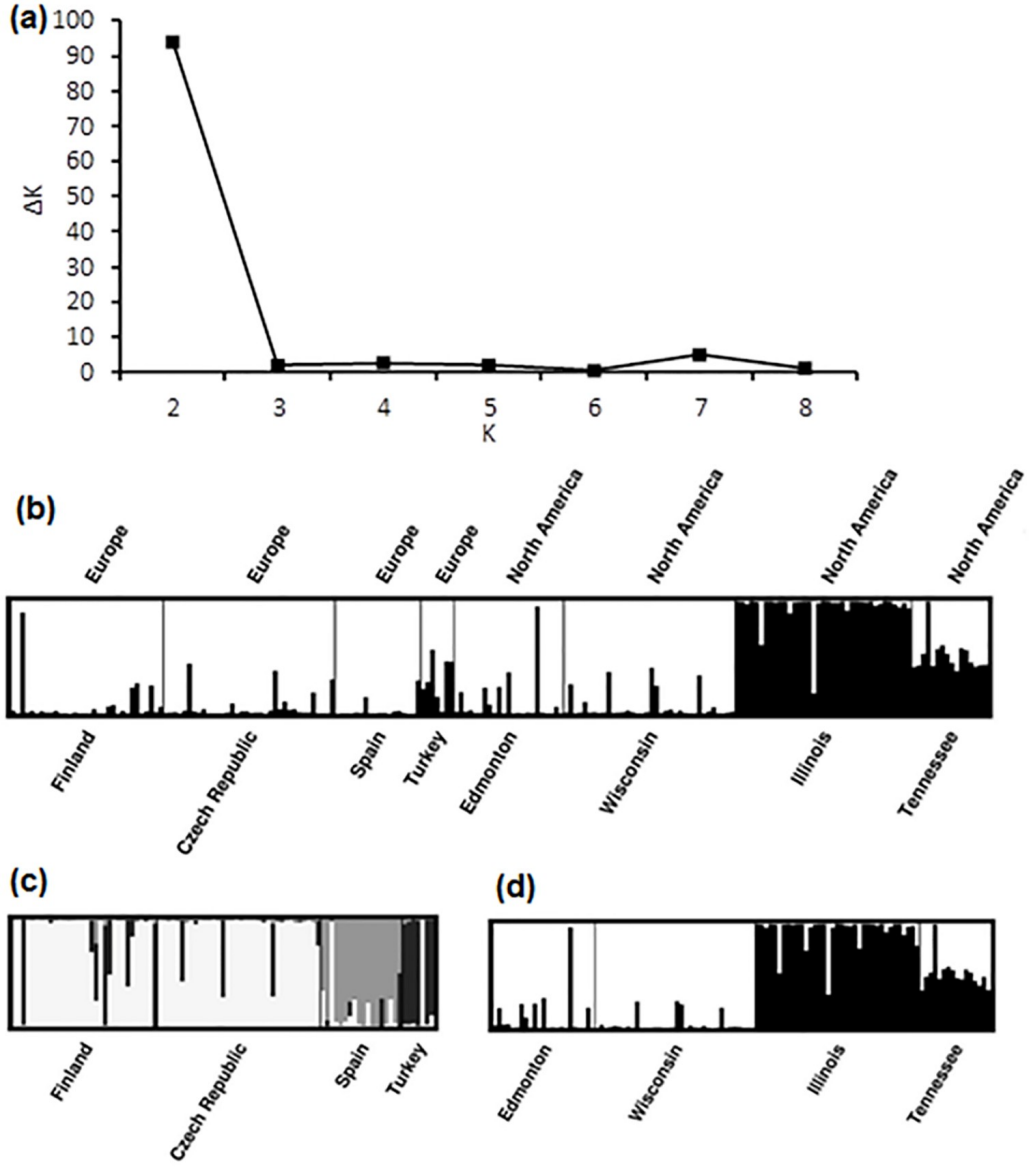
**Genetic similarity of *Lythrum salicaria* individuals germinated from seeds collected along two latitudinal gradients in Eurasia and North America** as determined using the program STRUCTURE: (a) a plot of the range of possible genetically distinct *L*. *salicaria* clusters, *K*, against *ΔK*, a model choice criterion for selection of the most likely *K*; (b); probabilistic assignment of *Lythrum salicaria* samples from populations along two latitudinal gradients to one of two genetically distinct clusters (white vs. black bars); (c) sub-comparisons for Eurasian populations (*K* = 3) and (d) North American populations (*K* = 2).

In exploring genetic differentiation among populations, two discrete population clusters were identified using the Bayesian clustering algorithms implemented in STRUCTURE (Fig 6). One cluster was represented almost entirely by samples collected in Illinois, while the other was comprised of Eurasian individuals, and those collected in the northern part of North America, including Edmonton and Wisconsin. Very little admixture was apparent between clusters except among the samples collected from the southernmost North American site in Tennessee. There was also some suggestion of admixture in samples from Turkey (five of seven samples). Overall, the *F*_*ST*_ from population comparisons was 0.242 (p < 0.0001 based on Fisher’s Exact Test). *F*_*ST*_ comparisons with Illinois were high, with some values reaching 0.320 (S3 Table).

As a follow-up to our initial STRUCTURE analysis, we conducted continental-level analyses using STRUCTURE to reveal any population structure masked by the pronounced separation of Illinois populations from others (Fig 6). This analysis revealed the presence of distinct population clusters in Eurasia, one cluster combining Finland and the Czech Republic, and the other Spain and Turkey (Fig 6). Not surprisingly, the pattern of separation in North America was largely unchanged from the larger analysis, with Illinois forming its own distinct population, Edmonton and Wisconsin clustering together into a second population, and Tennessee appearing as a group of admixed individuals (Fig 6).

## Discussion

### Morphological differences in cold vs. hot gardens

A wide variety of morphological responses and plastic variation of species to environment may be key indicators of invasiveness [61]. For *L*. *salicaria* in this study, we found that populations from different latitudes had varied responses to cold versus hot garden environments in Třeboň vs. Lafayette, respectively. Plants from populations from both Eurasia and North America grown in the cold garden had lower total biomasses from seeds from populations collected from the north of the range in Year 2, switching to the middle of the range by Year 3 (e.g., U-shaped distribution), so that the responses were not uniform from populations across latitudes (Table 2D; Fig 1H). Also, in the cold garden, seeds originating from the middle of the range had higher inflorescence biomasses than elsewhere, especially in Year 2 (hump-shaped distribution; Table 2C; Fig 1F). We did not find evidence of population-level differences in how maternal families responded to cold versus hot gardens (p > 0.05; Table 2A–2D). This lack of statistical difference among populations may have been related to the wide variance in phenotypic responses among maternal families (see Reaction Norms: Figs 2 and 3).

Our findings are not similar to a Danish common garden study of invasive *Impatiens glandiflora*, in which northern populations had lower total biomass than southern ones [62]. *Hypericum perforatum* from both introduced and native plants had higher performance if originally from northern versus southern environments [63]. Not similar to either of these studies, our study showed that *L*. *salicaria* plants generally had higher total biomass in colder environments regardless of their origin (Fig 1A–1H). Our common garden and field observation study shared at least one similarity, which was that above-ground biomass and heights were larger from plants in the southern part of the range. In the common garden study, the larger biomass of plants of southern provenance was true whether grown in the cold/dry or hot/wet common garden (Table 2 and Fig 1). Following these analyses, Hypothesis 1 was supported in that the patterns of tissue biomass did depend on the latitude of seed origin in both cold and hot garden settings. Overall, our results suggest that *L*. *salicaria* is a “northern species” and may not respond as well to southern climates, which could impact its persistence and invasion success in hotter climates. Latitudinal variation further suggests the evolution of larger ecotypes in the south of its range, perhaps as a mechanism for offsetting heat stress.

Field-measured plants of *L*. *salicaria* in Eurasia were taller than in North America north of 40°N latitude (Fig 4), but Eurasian plants declined in height with decreasing latitude, while North American plants did not. Thus, the latitudinal ecotypes typical of native *L*. *salicaria* may have been largely erased during the introduction of the species into North America. What is more, the existence of larger plants in the southern portions of North America may confer heightened competitive ability, as suggested by the Evolution of Increased Competitive Ability (EICA) hypothesis [16], although the evidence from our field analysis is at best circumstantial. Recent meta-analyses suggest that biotic interactions may be important as selective forces in invasive species dynamics (e.g., an ability to favor soil biota useful to regenerating individuals), but not only with respect to herbivores and competitors as suggested by EICA [64].

Previous work indicates that *L*. *salicaria* inflorescences are genetically predisposed toward smaller sizes and earlier flowering in northern populations compared to their southern counterparts (e.g., [30–31, 33] but our results showed inflorescence limitations in both northern and southern populations, particularly in the Třeboň garden during Year 2 (Fig 1F). Limited flowering has been proposed as an adaptive feature consistent with a relatively short growing season at high latitudes [30]. Interestingly, the relationship that we observed at the population level was related to garden location and not continent of seed origin, suggesting that local adaptation may not have been as important as phenotypic plasticity in the changing responses of this invasive species of North America. Overall, Hypothesis 4 was thus not supported because flowering time was not earlier in plants established from maternal plants from northern populations in both Eurasia and North America growing in both gardens. All plants grown in Lafayette consistently flowered later than in Třeboň (S3 Fig).

### Genetic differences and adaptation of global populations

Genetic diversity of species in invaded vs. native continents can give some insight into the patterns of morphology that we observed in the cold versus hot garden environments. One important finding of our study is that genetic diversity in many respects is similar in the Eurasian and North American populations of *L*. *salicaria*, regardless of continent and latitude of origin (S4 Table). Nevertheless, native populations of *L*. *salicaria* have higher overall heterozygosity in northern Eurasia, presumably because this cold-adapted species faces selection pressures that constrain heterozygosity to the south (e.g., hot temperatures; Table 4 and Fig 5). Therefore, our study did not fully support Hypothesis 3 regarding the predicted similarity of genetic diversity among populations because this factor differed across the latitudinal gradient in both Eurasia and North America. Populations of this invasive species in North America have higher heterozygosity in the south. The situation suggests either a history of more frequent introductions and thus greater genetic admixture in southern North America, or that populations invading North America were mostly drawn from southern provenances in Eurasia, reducing the stress they experienced in their introduced range and allowing them to retain more of their initial diversity (Fig 5).

The reaction norm outcomes of our study further illuminate underlying genetic effects. Regardless of population or continent of origin (p > 0.05), most maternal families exhibited plasticity for total biomass, with plants nearly always growing smaller under hot conditions in Lafayette (Fig 2). This effect was most pronounced at the southern extremes of the species range in both Eurasia (Turkey) and North America (Tennessee), and was also quite pronounced at the northern extreme in Eurasia (Finland). These results show that *L*. *salicaria* has maintained phenotypic plasticity throughout its range on both continents, exhibiting the greatest plasticity in the harshest environments where it is most adaptively advantageous. Plasticity for inflorescence biomass also showed substantial plasticity, but was much more variable among populations and years. Inflorescence biomass was generally greater in the cold garden, except in Year 1 when several populations (Turkey, Tennessee, Wisconsin), exhibited greater biomass in the hot garden. The latter suggests that some populations may have evolved to respond to heat stress by ramping up reproductive output in their first year when mortality may be at its highest level. These findings do not support Hypothesis 3 in that phenotypic expression differed in plants from seeds of maternal plants collected across the latitudinal range in Eurasia and North America.

Because of the similar levels of genetic diversity in many respects between native and non-native ranges, our study gives limited evidence that founding events did not produce a bottleneck effect in North America. This level of genetic diversity in North America could have been supported by historical genetic admixture owing to multiple introductions in North America. *Lythrum salicaria* also has a habit of obligate outcrossing, which could promote the retention of high genetic diversity under certain conditions (as suggested for another species by [65]). Our results are in general agreement with earlier studies [22–23], but most comparable to [24], which also employed hypervariable AFLP markers. Whereas [24] sampled over 2–5 degrees of latitude, our study covered 18–24 degrees. Chun *et al*., [24] also found no statistical differences in genetic diversity between originating continents with the exception of a single population in North America, which exhibited unusually low diversity. Therefore, both [24] and our study suggest that *L*. *salicaria* likely escaped a genetic bottleneck (see [10]), which is almost undoubtedly the case because of its intentional and repeated introduction by the horticultural industry [37]. In the end, the genetic variation displayed by a species in its introduced environment is likely to be related to both evolutionary history and chance [66].

Turning to genetic diversity at the metapopulation level, all *L*. *salicaria* populations in our study were significantly differentiated from each other (S4 Table), with populations no less differentiated within continents of origin than between them. The only regional genetic structure we detected related to the highly differentiated nature of a single North American population in Illinois, and the flow of genes from this population into a population in Tennessee. Most likely, this population appeared highly distinct from all Eurasian populations simply because of sampling limitations—further sampling would likely reveal its source population somewhere in Eurasia not encompassed by our initial survey. Nonetheless, our genetic comparisons among populations bolster the idea of repeated introductions into North America creating the potential for admixture, which would tend to fuel the adaptive potential of *L*. *salicaria* and contribute to its invasiveness.

From a management perspective, invasive *L*. *salicaria* of northern North America is likely to be adaptive to changing environments northward. We doubt that it will establish widely southward of its current distribution unless conditions become consistently wetter, noting that climate projections may predict extended drought in the future for this region in North America [67]. In the south, *L*. *salicaria* may ultimately be limited by water stress, which could be related to its reduced water-use efficiency at relatively high temperatures (e.g. (5)]). Near Venice Louisiana, *L*. *salicaria* grows in unleveed outfall areas near the mouth of the Mississippi River, where conditions may be nearly always flooded or at least moist near the soil surface (personal communication, Michael Massimi). Notably, horticulturalists in Louisiana (USA) have reported that *L*. *salicaria* grows well for a year or two, followed by declining performance (W. Fontenot, personal communication); we observed a similar decline in Louisiana plants by the third year of the garden study. Nevertheless, *L*. *salicaria* undoubtedly derives some of its invasive qualities from its plastic responses for tissue allocation across latitudes and between continents. These traits have likely conferred superior adaptive traits to *L*. *salicaria* for tolerating changing wetland environments.

## Supporting information

S1 Table Worldwide purple loosestrife volunteer collection program. (DOCX)

S2 Table Genotyping summary for eight populations of *Lythrum salicaria* grown from seed in a common garden study and compared via AFLP markers. Seed collection locations in the common garden studies included continent of origin, geographic location and place name. Genetic diversity statistics are given and abbreviated as: <P> = Proportion of polymorphic markers, <H> = Nei’s gene diversity, and PIC = Polymorphism information content.
(DOCX)

S3 Table Pairwise *F*_*ST*_ estimates (below diagonal) among eight *Lythrum salicaria* populations from Eurasian and North America (associated p-values appear above the diagonal). (DOCX)

S4 Table ANCOVA results for each of three genetic diversity variables measured on eight *L*. *salicaria* populations from Eurasian (native) and North America (invasive). Each ANOVA evaluated the main effects of continent of origin, latitude, and the interaction between continent and latitude. Significant p-values (p < 0.05) are indicated with an asterisk. Degrees of freedom for all effects were 1 and 4.
(DOCX)

S1 Fig Ordination graphs based on Principal Components Analysis depicting relationship of maternal responses of *Lythrum salicaria* plants as related to geographic seed origin from native Eurasian and invasive North American populations grown in gardens in Třeboň Czech Republic vs. Lafayette Louisiana (cold vs. hot; TR vs. LA, respectively) in 2006–2008. Maternal plants were grown from seeds collected along latitudinal gradients in Eurasia (Finland, Czech Republic, Spain and Turkey; FIN, CZ, SP and TK, respectively) and North America (Edmonton, Wisconsin, Illinois, and Tennessee; ED, WI, IL, and TN). Axis 1 indicated environmental differences between the two common gardens (variance explained 2006, 2007, 2008: 31.20, 43.49 and 43.73%, respectively). Axis 2 separated the populations in both gardens by latitude of population origin (variance explained 2006, 2007, 2008: 26.93, 21.83 and 23.25% respectively.
(DOCX)

S2 Fig Ordination graphs depicting centroids from Principal Components Analysis showing the overall responses of *Lythrum salicaria* plants as related to location of seed collection location of maternal plants from native Eurasian and invasive North American populations grown in gardens in Třeboň Czech Republic vs. Lafayette Louisiana (cold vs. hot; TR vs. LA, respectively) in 2006–2008. See S1 Fig for abbreviation key and variance explained.
(DOCX)

S3 Fig Strength of the relationship of the variables to environments represented by Axis 1 and 2. The graphs from Principal Components Analysis are based on the overall responses of *Lythrum salicaria* plants as related to location of seed collection maternal plants from native Eurasian and invasive North American populations grown in gardens in Třeboň Czech Republic vs. Lafayette Louisiana (cold vs. hot; TR vs. LA, respectively) in 2006–2008. See Appendix 5 for variance explained by Axis 1 and 2. Variable abbreviations include: time in days from start of growing season to harvest i.e. time to flowering (GrowTime), plant height (Stem Ht), number of shoots from the rootstock (Stem No), aboveground mass in grams dry biomass (Above DW), belowground mass in grams dry biomass (Root DW), aboveground biomass + below ground biomass DW (Total DW), root-to-shoot dry biomass ratio (RS), growth of plant measured as change in shoot height per day (Ht/Day), shoot dry biomass/ totalDW (shoot biomass ratio or SWR; includes leaf DW in 2008, leaf DW / totalDW (leaf biomass ratio or LWR; includes SWR for 2008), inflorescence DW / totalDW (reproductive effort or RE), and root DW / total DW (root biomass ratio or RWR).
(DOCX)
