# Supplementary material for: Morphology and genetics of Lythrum salicaria from latitudinal gradients of the Northern Hemisphere grown in cold and hot common gardens
Source: PLoS One. 2019 Jan 3;14(1):e0208300. doi: 10.1371/journal.pone.0208300 (PMC6317810; doi:10.1371/journal.pone.0208300)
Supplement: S1 Table — (DOCX) [file pone.0208300.s001.docx]

**Supporting Information**

**S1 Table. Worldwide purple loosestrife volunteer collection program.**

A worldwide group of volunteers participated in the measurement of height of *Lythrum salicaria*. These twenty-nine volunteers were recruited via the Purple Loosestrife Volunteer website (Middleton 2004), or as part of workshops. In the case of volunteers recruited via the internet, the methodology for the work was discussed by email; as part of workshops, one of the authors accompanied the volunteers in the field. In some cases, there were no volunteers for a particular region (e.g., northern Finland), so specific people were solicited to make measurements. Heights from Australia were eliminated because of too few geographic measurements within that subcontinent.

The volunteers collected data for the project in Australia, North America and Eurasia (A,N, E, respectively) and included: Paul Adam (A), J. P. Anderson (N), Nuket Akanil Bingol (E), Jenny Carol (N), Jack Carter (N), Malavika Chauhan (E), Katie Clark (N), Paul Cook (N), Kari Foster Cretini (E & N), Donna Devlin (N), David Hamilla (N), Ben Handley (N), Dallas Holland (N), Jan Kvet (E), Heather Lynn Lindon (N), Christi Maki (N), Robin Maerklein (N), Joy Marburger (N), Eva Middleton (N), Rudy Runshek (N), Raja Sengupta (N), Wendy Smith (N), Donna Stevens (N), Kathryn Theiss (N), Petra Vavlova (E), Kip Welton (N), Teresa Walte, Guodong Wang (E), and Terry Williard (N).
