# Supplementary material for: Morphology and genetics of Lythrum salicaria from latitudinal gradients of the Northern Hemisphere grown in cold and hot common gardens
Source: PLoS One. 2019 Jan 3;14(1):e0208300. doi: 10.1371/journal.pone.0208300 (PMC6317810; doi:10.1371/journal.pone.0208300)
Supplement: S2 Table — Seed collection locations in the common garden studies included continent of origin, geographic location and place name. Genetic diversity statistics are given and abbreviated as: = Proportion of polymorphic markers, = Nei’s gene diversity, and PIC = Polymorphism information content. (DOCX) [file pone.0208300.s002.docx]

**S2 Table. Genotyping summary for eight populations of *Lythrum salicaria* grown from seed in a common garden study and compared via AFLP markers.** Seed collection locations in the common garden studies included continent of origin, geographic location and place name. Genetic diversity statistics are given and abbreviated as: <P> = Proportion of polymorphic markers, <H> = Nei’s gene diversity, and PIC = Polymorphism information content.

| Continent of seed origin | Geographic location | Place Name | Latitude | Longitude | <P> | <H> | PIC |
| --- | --- | --- | --- | --- | --- | --- | --- |
| Eurasia | Finland | Vantaa River | 60^o^ 36’N | 21^o^ 26’ E | 63.1 | 0.211 | 0.170 |
|  | Czech Republic | Branišov | 48^o^ 59’N | 14^o^ 24’ E | 57.2 | 0.168 | 0.137 |
|  | Spain | Segre River | 41^o^ 37’N | 0^o^ 37’ E | 56.2 | 0.184 | 0.130 |
|  | Turkey | Antalya | 36^o^ 52’N | 31^o^ 11’ E | 65.4 | 0.218 | 0.153 |
| North America | Edmonton | Wabamun Lake | 53^o^ 33’N | 114^o^ 31’ W | 59.8 | 0.199 | 0.160 |
|  | Wisconsin | Okee | 43^o^ 21’N | 89^o^ 34’ W | 55.9 | 0.187 | 0.148 |
|  | Illinois | Arthur | 39^o^ 42’N | 88^o^ 28’ W | 64.7 | 0.207 | 0.185 |
|  | Tennessee | Nickajack | 35^o^ 00’N | 85^o^ 37’ W | 65.0 | 0.211 | 0.172 |
