# Supplementary material for: Morphology and genetics of Lythrum salicaria from latitudinal gradients of the Northern Hemisphere grown in cold and hot common gardens
Source: PLoS One. 2019 Jan 3;14(1):e0208300. doi: 10.1371/journal.pone.0208300 (PMC6317810; doi:10.1371/journal.pone.0208300)
Supplement: S3 Table — (DOCX) [file pone.0208300.s003.docx]

**S3 Table. Pairwise *F_ST_* estimates (below diagonal) among eight *Lythrum salicaria* populations from Eurasian and North America (associated p-values appear above the diagonal).**

|  |  |  |  | Site |  |  |  |  |
| --- | --- | --- | --- | --- | --- | --- | --- | --- |
|  | Finland | Czech Republic | Spain | Turkey | Edmonton | Wisconsin | Illinois | Tennessee |
| Finland | -- | <0.0001 | <0.0001 | <0.0001 | <0.0001 | <0.0001 | <0.0001 | <0.0001 |
| Czech Republic | 0.129 | -- | <0.0001 | <0.0001 | <0.0001 | <0.0001 | <0.0001 | <0.0001 |
| Spain | 0.175 | 0.167 | -- | <0.0001 | <0.0001 | <0.0001 | <0.0001 | <0.0001 |
| Turkey | 0.200 | 0.217 | 0.249 | -- | <0.0001 | <0.0001 | <0.0001 | <0.0001 |
| Edmonton | 0.150 | 0.151 | 0.202 | 0.177 | -- | <0.0001 | <0.0001 | <0.0001 |
| Wisconsin | 0.227 | 0.241 | 0.273 | 0.196 | 0.166 | -- | <0.0001 | <0.0001 |
| Illinois | 0.286 | 0.300 | 0.322 | 0.210 | 0.256 | 0.299 | -- | <0.0001 |
| Tennessee | 0.203 | 0.199 | 0.246 | 0.145 | 0.186 | 0.223 | 0.165 | -- |
