# Supplementary material for: Morphology and genetics of Lythrum salicaria from latitudinal gradients of the Northern Hemisphere grown in cold and hot common gardens
Source: PLoS One. 2019 Jan 3;14(1):e0208300. doi: 10.1371/journal.pone.0208300 (PMC6317810; doi:10.1371/journal.pone.0208300)
Supplement: S4 Table — Each ANOVA evaluated the main effects of continent of origin, latitude, and the interaction between continent and latitude. Significant p-values (p < 0.05) are indicated with an asterisk. Degrees of freedom for all effects were 1 and 4. (DOCX) [file pone.0208300.s004.docx]

**S4 Table. ANCOVA results for each of three genetic diversity variables measured on eight *L. salicaria* populations from Eurasian (native) and North America (invasive).** Each ANOVA evaluated the main effects of continent of origin, latitude, and the interaction between continent and latitude. Significant p-values (p < 0.05) are indicated with an asterisk. Degrees of freedom for all effects were 1 and 4.

| Genetic Diversity Variable | Source | F | P |
| --- | --- | --- | --- |
| Proportion of Polymorphic Loci | Continent | 0.011 | 0.9202 |
|  | Latitude | 0.241 | 0.6495 |
|  | Continent x latitude | 0.550 | 0.4997 |
| Nei’s Gene Diversity | Continent | 0.086 | 0.7842 |
|  | Latitude | 0.060 | 0.8183 |
|  | Continent x latitude | 0.118 | 0.7487 |
| Polymorphism Information Content | Continent | 2.565 | 0.1845 |
|  | Latitude | 0.099 | 0.7684 |
|  | Continent x latitude | 1.937 | 0.2364 |
