# Supplementary material for: Morphology and genetics of Lythrum salicaria from latitudinal gradients of the Northern Hemisphere grown in cold and hot common gardens
Source: PLoS One. 2019 Jan 3;14(1):e0208300. doi: 10.1371/journal.pone.0208300 (PMC6317810; doi:10.1371/journal.pone.0208300)
Supplement: S1 Fig — Maternal plants were grown from seeds collected along latitudinal gradients in Eurasia (Finland, Czech Republic, Spain and Turkey; FIN, CZ, SP and TK, respectively) and North America (Edmonton, Wisconsin, Illinois, and Tennessee; ED, WI, IL, and TN). Axis 1 indicated environmental differences between the two common gardens (variance explained 2006, 2007, 2008: 31.20, 43.49 and 43.73%, respectively). Axis 2 separated the populations in both gardens by latitude of population origin (variance explained 2006, 2007, 2008: 26.93, 21.83 and 23.25% respectively. (DOCX) [file pone.0208300.s005.docx]

**S1 Fig. Ordination graphs based on Principal Components Analysis depicting relationship of maternal responses of *Lythrum salicaria* plants as related to geographic seed origin from native Eurasian and invasive North American populations grown in gardens in Třeboň Czech Republic vs. Lafayette Louisiana (cold vs. hot; TR vs. LA, respectively) in 2006-2008.** Maternal plants were grown from seeds collected along latitudinal gradients in Eurasia (Finland, Czech Republic, Spain and Turkey; FIN, CZ, SP and TK, respectively) and North America (Edmonton, Wisconsin, Illinois, and Tennessee; ED, WI, IL, and TN). Axis 1 indicated environmental differences between the two common gardens (variance explained 2006, 2007, 2008: 31.20, 43.49 and 43.73%, respectively). Axis 2 separated the populations in both gardens by latitude of population origin (variance explained 2006, 2007, 2008: 26.93, 21.83 and 23.25% respectively
