# Supplementary material for: Morphology and genetics of Lythrum salicaria from latitudinal gradients of the Northern Hemisphere grown in cold and hot common gardens
Source: PLoS One. 2019 Jan 3;14(1):e0208300. doi: 10.1371/journal.pone.0208300 (PMC6317810; doi:10.1371/journal.pone.0208300)
Supplement: S2 Fig — See S1 Fig for abbreviation key and variance explained. (DOCX) [file pone.0208300.s006.docx]

**S2 Fig. Ordination graphs depicting centroids from Principal Components Analysis showing the overall responses of *Lythrum salicaria* plants as related to location of seed collection location of maternal plants from native Eurasian and invasive North American populations grown in gardens in Třeboň Czech Republic vs. Lafayette Louisiana (cold vs. hot; TR vs. LA, respectively) in 2006-2008.** See S1 Fig for abbreviation key and variance explained.
