# Supplementary material for: Morphology and genetics of Lythrum salicaria from latitudinal gradients of the Northern Hemisphere grown in cold and hot common gardens
Source: PLoS One. 2019 Jan 3;14(1):e0208300. doi: 10.1371/journal.pone.0208300 (PMC6317810; doi:10.1371/journal.pone.0208300)
Supplement: S3 Fig — The graphs from Principal Components Analysis are based on the overall responses of Lythrum salicaria plants as related to location of seed collection maternal plants from native Eurasian and invasive North American populations grown in gardens in Třeboň Czech Republic vs. Lafayette Louisiana (cold vs. hot; TR vs. LA, respectively) in 2006–2008. See Appendix 5 for variance explained by Axis 1 and 2. Variable abbreviations include: time in days from start of growing season to harvest i.e. time to flowering (GrowTime), plant height (Stem Ht), number of shoots from the rootstock (Stem No), aboveground mass in grams dry biomass (Above DW), belowground mass in grams dry biomass (Root DW), aboveground biomass + below ground biomass DW (Total DW), root-to-shoot dry biomass ratio (RS), growth of plant measured as change in shoot height per day (Ht/Day), shoot dry biomass/ totalDW (shoot biomass ratio or SWR; includes leaf DW in 2008, leaf DW / totalDW (leaf biomass ratio or LWR; includes SWR for 2008), inflorescence DW / totalDW (reproductive effort or RE), and root DW / total DW (root biomass ratio or RWR). (DOCX) [file pone.0208300.s007.docx]

**S3 Fig. Strength of the relationship of the variables to environments represented by Axis 1 and 2.** The graphs from Principal Components Analysis are based on the overall responses of *Lythrum salicaria* plants as related to location of seed collection maternal plants from native Eurasian and invasive North American populations grown in gardens in Třeboň Czech Republic vs. Lafayette Louisiana (cold vs. hot; TR vs. LA, respectively) in 2006-2008. See Appendix 5 for variance explained by Axis 1 and 2. Variable abbreviations include: time in days from start of growing season to harvest i.e. time to flowering (GrowTime), plant height (Stem Ht), number of shoots from the rootstock (Stem No), aboveground mass in grams dry biomass (Above DW), belowground mass in grams dry biomass (Root DW), aboveground biomass + below ground biomass DW (Total DW), root-to-shoot dry biomass ratio (RS), growth of plant measured as change in shoot height per day (Ht/Day), shoot dry biomass/ totalDW (shoot biomass ratio or SWR; includes leaf DW in 2008, leaf DW / totalDW (leaf biomass ratio or LWR; includes SWR for 2008), inflorescence DW / totalDW (reproductive effort or RE), and root DW / total DW (root biomass ratio or RWR).
